# Supplementary material for: Revision Arthroscopic Bankart Repair: A Systematic Review of Clinical Outcomes
Source: J Clin Med. 2020 Oct 25;9(11):3418. doi: 10.3390/jcm9113418 (PMC7693917; doi:10.3390/jcm9113418)
Supplement: Supplementary file 1 [file jcm-09-03418-s001.pdf]

Supplementary Files

Table S1. Patient characteristics.

Authors	Number of Patients in Study	Number of Shoulders in Study	Ages (years)	Gender Ratio (M:F)	Dominant Limb (Dominant:Non-dominant)	Follow-up Periods (months)
Arce et al.	16	16	26.8 (16-42)	13:3	11:5	30.9 (24-46)
Balazs et al.	16	16	20.9 (18-24)	12:4	10:6	32.8 (12-60)
Barnes et al.	16	17	30.0 (17-55)	13:3	7:10	38 (24-67)
Bartl et al.	56	56	29.4 (18-51)	45:11	40:16	37 (25-72)
Buckup et al.	23	23	25.5 ± 7.27	23:0	14:9	28.7 ± 8.45
Creighton et al.	18	18	28.6 (15-50)	15:3	9:9	29.7 (24-48)
Franceschi et al.	10	10	25.6 (18-41)	8:2	9:1	68 (46-83)
Kim et al.	23	23	24 (17-34)	21:2	19:4	36 (24-52)
Krueger et al.	20	20	29 (16-45)	19:1	11:9	24.7 (19-35)
Millar et al.	10	10	35 (23-55)	10:0	7:3	37 (12-89)
Neri et al.	12	12	28 (18-56)	10:2	7:5	34.4 (25-56)
Patel et al.	40	40	33.1 (15-48)	34:6	11:29	36 (12-87)
Ryu et al.	15	15	27.5 (17-44)	12:3	12:3	22 (18-70)
Shin et al.	62	63	23.2 (14.7-47.2)	46:16	34:29	46.9 (18-78)

M, male; F, female.

Table S2. Preoperative factors of revision surgery.

Authors	Poor Technique	Trauma	Glenoid Loss	Humeral Loss	Hyperlaxity
Arce et al.	12 (75%)	4 (25%)	NA	NA	Positive anterior apprehension sign: 3 (19%)
Balazs et al.	NA	NA	Calculated: 9% (0%-22%)<br>Observed: 5.3% (0%-20%)	Engaging Hill-Sachs: 0 (0%)	Positive anterior apprehension sign: 16 (100%)
Barnes et al.	NA	NA	NA	NA	Positive anterior apprehension sign: 3 (18%)
Bartl et al.	NA	43 (77%)	Total 22 (39%) checked<br><10%: 15 (27%)<br>11-20%: 7 (12%)	NA	Clinical sign (+): 16 (29%)
Buckup et al.	0 (0%)	3 (13%)	NA	NA	NA
Creighton et al.	NA	NA	NA	NA	NA
Franceschi et al.	NA	7 (70%)	NA	Total 6 (60%) checked<br>Calandra<br>Grade I: 1 (10%)<br>Grade II: 2 (20%)<br>Grade III: 3 (30%)	Anterior translation: 10 (100%)<br>Grade 2: 3 (30%)<br>Grade 3: 7 (70%)
Kim et al.	5 (22%)	9 (39%)	Total 14 (60%) checked<br><10%: 7 (30%)<br>11-20%: 4 (17%)<br>21-30%: 3(13%)	Total 23 (100%) checked<br>Calandra<br>Grade I: 1 (4%)<br>Grade II: 2 (9%)<br>Grade III: 20 (87%)	Anterior translation: 23 (100%)<br>Grade 2: 3 (13%)<br>Grade 3: 20 (87%)<br>Inferior translation: 3 (13%)<br>Grade 1: 2 (9%)<br>Grade 2: 1 (4%)
Krueger et al.	NA	NA	NA	Total 20 (100%) checked<br>Calandra<br>Grade I: 3 (15%)<br>Grade II: 9 (45%)<br>Grade III: 8 (40%)	NA
Millar et al.	0 (0%)	10(100%)	Total 5 (50%) checked<br>Mild: 4 (40%)<br>Severe: 1 (10%)	Total 6 (60%) checked<br>Mild: 5 (50%)<br>Severe: 1 (10%)	NA
Neri et al.	6 (50%)	2(17%)	NA	Total 9 (75%) checked<br>Calandra<br>Grade I: 7 (58%)<br>Grade III: 2 (17%)	Anterior translation: 12 (100%)<br>Grade 2: 8 (67%)<br>Grade 3: 4 (33%)<br>Posterior translation: 5 (41%)<br>Grade 2: 1 (8%)<br>Sulcus: 4 (33%)
Patel et al.	NA	32(80%)	Total 5(13%) checked	Total 17(43%) checked	Anterior translation: 34 (85%)<br>Grade 1: 8 (20%)<br>Grade 2: 17 (42.5%)<br>Grade 3: 9 (23.5%)

					Posterior translation: 1 (2.5%)<br>Grade 2: 1 (2.5%)<br>Multidirectional instability: 3 (8%)
Ryu et al.	NA	NA	Total 5 (33%) checked<br><10%: 1 (7%)<br>11-15%: 2 (13%)<br>>20%: 2 (13%)	Total 13 (86%) checked<br>Small Hill-Sachs: 8 (53%)<br>Engaging Hill-Sachs: 5 (33%)	Sulcus sign (+): 3 (20%)
Shin et al.	NA	NA	NA	NA	Clinical sign (+): 13(21%)
Total	23/84	110/190	51/144	94/146	Anterior: 130/253<br>Posterior: 6/52<br>Inferior: 6/38<br>Multidirectional: 3/40

NA: Not available.

Table S3. Clinical outcomes and range of motion after revision surgery.

Authors	Rowe Score	SST	UCLA Score	ASES Score	Constant Score	SANE Score	WOSI Score	Walch-Duplay Score	Subjective Shoulder Value Score	Visual Analog Pain Scale	Melbourne Instability Shoulder Score	Pre-operative ROM	Final Follow-up ROM
Arce et al.	33→80	-	22→31	-	69→80	-	-	-	-	-	-	NA	FF: 160 (135-180)<br>AB: 85 (70-100)<br>ER in 0° AB: 45 (15-75)<br>Loss of ER : 15 (0-35)<br>ER in 90° AB: 80 (65-100)<br>IR in 90° AB: T9 (L1-T5)
Balazs et al.	-	*9.7→10.7	-	67.8→85	-	50.6→82.1	45.4%→68.9%	-	-	-	-	NA	NA
Barnes et al.	83.8	8.3→11.3	-	-	-	-	-	-	-	-	-	NA	NA
Bartl et al.	68→85	7→11	-	-	72→87	-	-	-	-	-	-	NA	NA
Buckup et al.	90	-	-	83	90	-	-	85.5	-	-	-	NA	ER in 90° AB deficit: 9.25<br>ER in 0° AB deficit: 12
Creighton et al.	-	6→10	-	50→76	-		-	-	-	6→2	-	NA	ER in 0° AB deficit: 5

Franceschi et al.	-	-	11.7→31.7	-	-	-	-	-	-	-	-	*FF: 160 (140-170)<br>*ER: 128 (90-140)<br>*IR: 41 (30-45)	*FF: 157 (140-170)<br>*ER: 130 (90-140)<br>*IR: 40 (30-45)
Kim et al.	87.3→91.2	8.3→11.0	24.8→33.2	-	-		-	-	-	-	-	ROM deficit<br>*FF: 5.4 ± 11<br>*ER in 0° AB: 6.9 ± 16<br>ER in 90° AB.: NA<br>*IR in 90° AB: 0.9 ± 2.1	ROM deficit<br>*FF: 6.0 ± 12<br>*ER in 0° AB: 7.0 ± 18<br>ER in 90° AB.: 7.6 ± 15<br>*IR in 90° AB: 0.8 ± 0.9
Krueger et al.	*89 vs. 81.75	-	-	-	-	91.78 vs. 69.25	89.84% vs. 68.85%	85.25 vs. 75.5	91.78% vs. 69.25%	-	90.18 vs. 73.7	FF: 177<br>AB: 179<br>*ER in 0° AB: 63<br>*Loss of ER: 10.8<br>*ER in 90° AB: 80.5<br>*IR in 90° AB: 77.5	FF: 173.8<br>AB: 169.3<br>*ER in 0° AB: 64.3<br>*Loss of ER: 7.3<br>*ER in 90° AB: 78.8<br>*IR in

Millar et al.	37→93	-	16→31	-	-	-	-	-	-	-	-	*FF: 176 *AB: 143 ER: 50 *IR: T11	90° AB: 76.3 *FF: 176 *AB: 174 ER: 82 *IR: T10
Neri et al.	74.5	11.45	29.6	-	-	-	-	-	-	-	-	ER: 64.1 ± 15.6	ER: 69.5 ± 17.4
Patel et al.	-	-	-	81.1	-	-	68.2%	-	-	-	-	NA	NA
Ryu et al.	-	-	-	-	-	86	-	-	-	-	-	NA	NA
Shin et al.	-	6.1→9.0	-	63.7→85.1	-	-	80.1%	-	-	2.89→0.81	-	NA	NA
Mean	63.94→85.26	7.11→10.33	20.32→31.57	61.83→82.76	71.33→86.54	50.60→78.21	45.40%→73.77%	80.5	69.25	3.58→1.07	73.7		

SST, Simple Shoulder Test; UCLA, University of California, Los Angeles; ASES, American Shoulder and Elbow Surgeons; SANE, Single Assessment Numeric Evaluation; WOSI, Western Ontario Shoulder Instability; * Data that has statistically no significance; c.f. Krueger et al.: Initial group vs. Revision group.
